# The Story of the Insane from Year to Year

**Published:** 1902-01-11

**Authors:** 


### THE STORY OF THE INSANE FROM YEAR TO YEAR.

(Thirteenth Series.)

### ROXBURGH DISTRICT ASYLUM.

At the beginning of the year this asylum contained 297 patients, and at the end of the year there were 303 in residence. There were 45 first admissions, 15 readmissions, 18 recoveries, 10 discharged relieved, and 10 deaths. The total number of persons under care during the year was 355, and the average daily number resident was 303. The recoveries stand at 30 per cent. of the admissions, and the deaths at 8·2 per cent. of the average number resident. The recoveries are considerably lower than the average of public asylums, and also below the Roxburgh average, while the deaths are below the general average, and considerably above the Roxburgh average, which is only 6·9 per cent. for the last five-and-twenty years. Cerebral disease caused only one death, and consumption only two during the year. Of the year's admissions, six are supposed to owe their insanity to moral causes, and as usual drink and hereditary influence are the most potent physical causes, the former being credited with 9 and the latter with 24. There were 28 private patients in the asylum at the end of the year, and the ordinary rate of board is only £40 per annum, but patients in straitened circumstances are received at even lower rates. Some of our English so-called charitable lunatic hospitals sound a clarion note of self-praise for their kindness when they receive patients at £50 a year; and we would that some voice would utter the doings of asylums like the Roxburgh to them. It might awaken them to a new life. Additions are being made to the asylum, and Dr. Johnstone says that good progress is being made with them. The annual rate of maintenance is £25 per head.

### IPSWICH BOROUGH ASYLUM.

On January 1st, 1899, this asylum contained 317 patients, and on December 31st the number had fallen to 297. There were 73 first admissions, 9 readmissions, 34 recoveries, 22 discharged relieved, 18 discharged not improved, and 28 deaths. The average daily number resident was 312, and the total number of patients under care was 397. Reckoned on the admissions the percentage of recoveries was 41·4 per cent., and reckoned on the average number resident the percentage of deaths was 9·2. Of the year's deaths 10 were caused by cerebral and spinal diseases, 6 by phthisis, 1 by pneumonia, and 1 by pleurisy. Of the admissions of the year it would seem that moral causes account for the insanity in only 4 cases, while of the physical causes intemperance in drink accounts for 4, "previous attacks" for 19, and hereditary influence for 16. The table showing the assigned causes of insanity is necessarily an unsatisfactory one. In some cases the union officials do not make very strict inquiries into the causation of the disease, and it does not always happen that the Asylum Medical Officers pursue these investigations after admission. Here Table X. is so carefully compiled that the cause of the insanity is stated in the whole number of the admissions, indeed we rather fancy we have one more than Table No. 1 would justify. The subject has evidently interested Dr. Rowe. As regards moral causes and alcoholic intemperance he thinks there may be more cases than the 8 attributed to them, in which these causes had something to do with the insanity; and he adds that "it seems anomalous that bodily disease was recorded in only 21 instances, whereas bodily ill-health is the cause of by far the greater proportion of cases of mental disease." In a certain sense this is true, at all events the majority of patients admitted to our county asylums are in feeble health; but it is equally true that serious ill-health rarely ends in insanity unless there be a more or less strong hereditary taint. A considerable number of out-county and private patients are accommodated in the Ipswich Asylum and constitute such a source of income that the committee handed over a sum of £2,000 to the Borough Fund. The weekly cost per head was 9s. 2d., but for some reason the Borough was charged 10s. 11d. The charge for out-county patients ranges from 14s. to 25s., and the charge for private patients also ranges from 14s. to 25s. a week. The committee say that "the management of the asylum, and the conduct of the officers and attendants, and the care of the patients, have been satisfactory."

### LONDON COUNTY ASYLUM AT BEXLEY HEATH.

This asylum contained 262 patients on January 1st, 1899, and by December 31st of the same year the number had risen to 1,174. There were 1,162 first admissions, 3 readmissions, 95 discharged recovered, 18 discharged relieved, 62 discharged not improved, and 78 deaths. The average number resident during the year was 704, and the total number under treatment was 1,427. As 1899 was the first year of the existence of the asylum it would not seem desirable to state any other statistics, as they would not be in any sense useful for comparison with other asylums. The difficulties attending the opening and organising of a large asylum are by no means slight, and Dr. Stansfield may congralute himself that no serious casualty or mishap marred his first year of office, and the Visiting Committee very truly say that much credit is due to him for the efficient state of the asylum. The weekly cost per head was 10s. 9d.

